# A 3D-Printed PLCL Scaffold Coated with Collagen Type I and Its Biocompatibility

**DOI:** 10.1155/2018/5147156

**Published:** 2018-02-28

**Authors:** Yong He, Wei Liu, Lianxiong Guan, Jielin Chen, Li Duan, Zhaofeng Jia, Jianghong Huang, Wencui Li, Jianquan Liu, Jianyi Xiong, Lijun Liu, Daping Wang

**Affiliations:** ^1^Department of Orthopedics, The First Hospital Affiliated to Shenzhen University, Shenzhen, Guangdong 518035, China; ^2^Shenzhen Key Laboratory of Tissue Engineering, The First Hospital Affiliated to Shenzhen University, Shenzhen, Guangdong 518035, China; ^3^Department of Orthopedics, People's Hospital of Yangjiang, Yangjiang, Guangdong 529500, China

## Abstract

Scaffolds play an important role in tissue engineering and their structure and biocompatibility have great influence on cell behaviors. In this study, poly(l-lactide-co-*ε*-caprolactone) (PLCL) scaffolds were printed by a 3D printing technology, low-temperature deposition manufacturing (LDM), and then PLCL scaffolds were treated by alkali and coated with collagen type I (COLI). The scaffolds were characterized by scanning electron microscopy (SEM), porosity test, mechanical test, and infrared spectroscopy. The prepared PLCL and PLCL-COLI scaffolds had three-dimensional (3D) porous structure and they not only have macropores but also have micropores in the deposited lines. Although the mechanical property of PLCL-COLI was slightly lower than that of PLCL scaffold, the hydrophilicity of PLCL-COLI was significantly enhanced. Rabbit articular chondrocytes were extracted and were identified as chondrocytes by toluidine blue staining. To study the biocompatibility, the chondrocytes were seeded on scaffolds for 1, 3, 5, 7, and 10 days. MTT assay showed that the proliferation of chondrocytes on PLCL-COLI scaffold was better than that on PLCL scaffold. And the morphology of cells on PLCL-COLI after 1-day culture was much better than that on PLCL. This 3D-printed PLCL scaffold coated with COLI shows a great potential application in tissue engineering.

## 1. Introduction

Cartilage defect caused by sports injury, inflammation, degeneration, and other reasons is a common disease in clinic. Due to the lack of blood vessels and nerves in cartilage tissue and chondrocytes being confined to a dense lacuna consisting of collagen and proteoglycans, the regeneration and self-repair ability of cartilage are extremely limited [1]. In clinic, current techniques for cartilage repair are varied and each has its own drawbacks [2–4]. Scaffold is one of three elements of tissue engineering, and the fabrication of ideal tissue engineering scaffold attracts a lot of attentions [5]. However, conventional fabrication technologies such as salt leaching, electrospinning, and fiber bonding cannot precisely control the pore size and structure of the scaffold [6, 7].

To fabricate three-dimensional (3D) scaffold with controlled pore size and structure, various novel technologies have been reported, especially the recently developed low-temperature deposition manufacturing (LDM) [8]. LDM, based on the principle of rapid prototyping technology, is characterized with personalized printing, simple operation, less waste, less pollution, and so on. It is a green manufacturing [9–11]. More importantly, it preserves the bioactivities of the materials due to its nonheating feature. Natural biopolymers such as collagen type I (COLI), gelatin, sodium alginate, and chitosan have been printed successfully by LDM without compromising their bioactivities [12–17]. Based on the computer-aided design data, LDM is employed to build scaffold layer by layer on a platform in a low-temperature chamber and then the scaffold is freeze-dried to remove the frozen solvent. LDM also combined rapid deposition manufacturing process with phase separation process. Besides the controlled macropore size, scaffold fabricated by LDM has interconnected micropores in the deposited lines due to the phase separation process, which can significantly increase the porosity of the scaffold and provide more topological cues for cells attachment [18].

COLI is a natural polymeric material and is one of the main component of connective tissues. Its safety, biocompatibility, hydrophilicity, pyrogenic immunogenicity make it suitable for tissue engineering applications. And its effect on satisfactory medical application has been confirmed and it has been widely used in surgical sutures, anticoagulation materials, artificial blood vessels, skin, cartilage repair, and so on [19–21]. However, collagen scaffold for cartilage tissue engineering presents obvious shortcomings such as low mechanical strength and fast degradability [22, 23]. As a synthetic polymeric material approved by the US FDA for clinical application, poly(l-lactide-co-*ε*-caprolactone) (PLCL) has excellent mechanical properties, high plasticity, and controlled degradation rate, but its poor hydrophilicity, low biocompatibility, and acid degradation products are noted. Many studies have shown that COLI incorporated in polycaprolactone, polylactic acid, PLCL, and other synthetic polymers can be used in tissue engineering and to promote cell adhesion and proliferation [24–28].

In this study, a porous 3D-printed PLCL scaffold coated with type I collagen was fabricated by LDM. PLCL could work as a mechanical support; on the other hand, COLI could improve the biocompatibility of PLCL. It is expected that PLCL-COLI scaffolds could combine the advantages of natural polymer materials and synthetic polymer materials. The physical and chemical properties and biocompatibility of PLCL-COLI composite scaffolds were investigated in order to provide theoretical and experimental support for their feasibility as an ideal scaffold for cartilage tissue engineering.

## 2. Experimental

### 2.1. Materials

PLCL (PLLA : PCL, 50 : 50; dl/g = 1.67; catalog number: 105736-876) was obtained from Jinan Daigang Biomaterials Co., China. COLI (*M*_*W*_ = 300,000) from porcine skin was bought from Sichuan Ming Rang Biological Technology Co., China. The solvent 1,4-dioxane (DIO) (catalog number: D0860) was purchased from Aladdin Co., China. All products were used without further purification.

### 2.2. Fabrication and Modification of the Scaffold

1 g PLCL material was dissolved in 10 mL DIO and stirred overnight under 600 RPM at room temperature to prepare a homogeneous solution. The PLCL scaffolds were fabricated by LDM system (Tissue Form II, Tsinghua University, China). The printing parameters are as follows: size is 2.4 × 2.4 × 2.4 cm^3^; molding temperature is within −25~−35°C; the nozzle diameter is 0.4 mm; spinning distance is 1.0 mm; scanning speed is within 15~30 mm/s; and the nozzle speed is 1.0~2.0 mm/s. After printing, the formed and frozen scaffold was taken out from the forming chamber and freeze-dried for 48 hours in a freeze-dryer (Beijing Bo Medical Experimental Instrument Co., China), where gas-solid phase separation process happened. Due to the phase separation process, interconnected micropores in the deposited lines were produced, and then the 3D porous PLCL scaffold was fabricated. The as-fabricated scaffold was treated for 10 min in 0.2 wt% NaOH solution and rinsed in DI water for several times to remove the residual NaOH to obtain alkaline-modified scaffold (aPLCL). The aPLCL scaffold was immersed in 0.5% (w/v) COLI acetic acid solution for 4 hours and then rinsed in PBS for several times to remove the unattached COLI and freeze-dried.

### 2.3. Morphology and Structure Observation

The as-prepared scaffolds were photographed by digital optical camera (Nikon D3100, Nikon Corporation). The morphology and structure of PLCL, aPLCL, and PLCL-COLI composite scaffolds were analyzed by scanning electron microscopy (SEM) (Hitachi, Japan). Samples were mounted on sample holders and placed in a SEM chamber with prior addition of a sputter coating. Digital images were captured. Pore size was analyzed by Image J software and the average pore size was calculated.

### 2.4. Porosity

The porosity of PLCL and PLCL-COLI composite scaffolds was determined using liquid replacement method. Ethanol can permeate the macropores and micropores without causing expansion or contraction of the scaffolds and could be used as a substitute for measurement [7]. The PLCL-COLI scaffolds were cut into several rectangular cubes (*n* = 5). The volume of every cube was measured and marked as *V*_1_. Then, the cubes were placed in a cylinder containing moderate ethanol with volume *V*_2_. The cylinder was placed in the vacuum pump until the cubes did not emit bubbles, which means that all the pores were filled with ethanol and marked the volume as *V*_3_. The porosity was calculated by the following equation: *ρ*(%) = (*V*_1_ + *V*_2_ − *V*_3_) × 100/*V*_1_.

### 2.5. Hydrophilicity

The PLCL and PLCL-COLI scaffolds were cut into cubes of 0.9 × 0.9 × 0.9 cm^3^, and the weight of the cubes was measured as *W*_1_ (*n* = 4). The cubes were soaked in distilled water and taken out after 3 days. Use the filter paper to dry the water on the surface of the cubes and weigh the mass as *W*_2_. The hydrophilicity = (*W*_2_ − *W*_1_)/*W*_2_  × 100%.

### 2.6. Mechanical Testing

The mechanical properties were assessed by Electronic Universal Testing Machine integrated testing software Material Test (Insrton, USA). The scaffolds were cut into 1 × 1 × 2 cm^3^ cubes (*n* = 4). Tests were carried out using a 100 N load at a velocity of 1 mm/min, up to a maximum of 70% strain. The compressive modulus was defined as the slope of a linear fit to the stress-strain curve over 0–5% strain [29, 30].

### 2.7. Infrared Test

Infrared tests of PLCL, PLCL-COLI scaffold, and COLI were carried out by Fourier Transform infrared spectroscopy (Thermo, USA), and the FTIR spectroscopy was analyzed.

### 2.8. Isolation and Identification of Rabbit Chondrocytes

A 6-week-old New Zealand female rabbit (Oryctolagus cuniculus) was injected with 3% pentobarbital sodium solution under general anesthesia. The articular cartilage tissue of nonweight-bearing knee joints was obtained under aseptic conditions. The cartilage tissue was rinsed with PBS and cut into small pieces. The pieces were placed in a 15 mL centrifuge tube, and then 5 mL collagenase solution was added. The tube was shaken at 37°C for 6 to 8 hours using a thermostat shaker. The digestive tissue fluid was filtered by a 100 *μ*m cell filter. The filtered solution was centrifuged at 1500 r/min for 10 min; the supernatant was discarded and the cell suspension was prepared by adding DMEM/F-12 (1 : 1) (LOT: 8806253, Gibco, USA) supplemented with 10% FBS and 1% penicillin-streptomycin to the precipitated cells. The rabbit articular cartilage chondrocytes were seeded into 25 cm^2^ cell culture dishes at 2 × 10^4^ cells/cm^2^ and cultured at 37°C in a 5% CO_2_ incubator (Thermo, USA). Media replacement was carried out every 72 h until the cells reached a 90% confluent layer. Cells were digested with 0.25% (w/v) trypsin plus 0.02% (w/v) EDTA (HyClone, USA) and subcultured at a density of 1.0 × 10^4^ cells/cm^2^. The rabbit chondrocytes of passage 2 were used for identification by toluidine blue. For toluidine blue experiment, cells were fixed with 4% (w/v) paraformaldehyde for 1 h and stained with 1% (w/v) toluidine blue dye.

### 2.9. Cell Viability Assay

The PLCL and PLCL-COLI scaffolds were sliced into rounds with diameter of 10.0 mm and thickness of 1 mm. The scaffolds were sterilized by immersing in 75% ethanol, rinsed, and placed in 24-well plates. The rabbit chondrocytes were seeded in the small disc scaffolds at 5 × 10^4^/mL, 1 mL per well. 1 mL DMEM/F-12 (1 : 1) supplemented with 10% FBS and 1% penicillin-streptomycin was added in per well. The culture plates were placed in the 37°C incubator supplied with 5% CO_2_. The media were refreshed every 3 days.

MTT assay was used to test the cell viability. Cells on scaffolds at days 1, 3, 5, 7, and 10 were used for the assay. In this experiment, media on the surface of the scaffolds were discarded, 500 *μ*L media was added to each well and 100 uL MTT (KeyGEN, China) was added per well and incubated at 37°C for 2 h. The absorbances were measured at 490 nm using a microplate reader (BioTek, Winooski, USA).

### 2.10. Cell Morphology

Cell density of 5 × 10^4^ cells/mL was seeded on PLCL and PLCL-COLI for 1 day, 5 days, and 10 days. Scaffolds with cells were fixed in 4% paraformaldehyde solution for 30 minutes at room temperature; then they were dehydrated with increasing concentrations of ethanol (50%, 70%, 95%, and 100%) for five cycles. Samples were subsequently air-dried overnight and observed by FE-SEM (TESCAN MIRA3).

### 2.11. Statistical Analysis

Data are presented as means ± standard deviation. Statistical comparisons were performed using Student's *t*-test. *P* values < 0.05 were considered significant.

## 3. Results and Discussion

### 3.1. Morphology and Structure of the Porous Scaffolds

**Figure 1:**
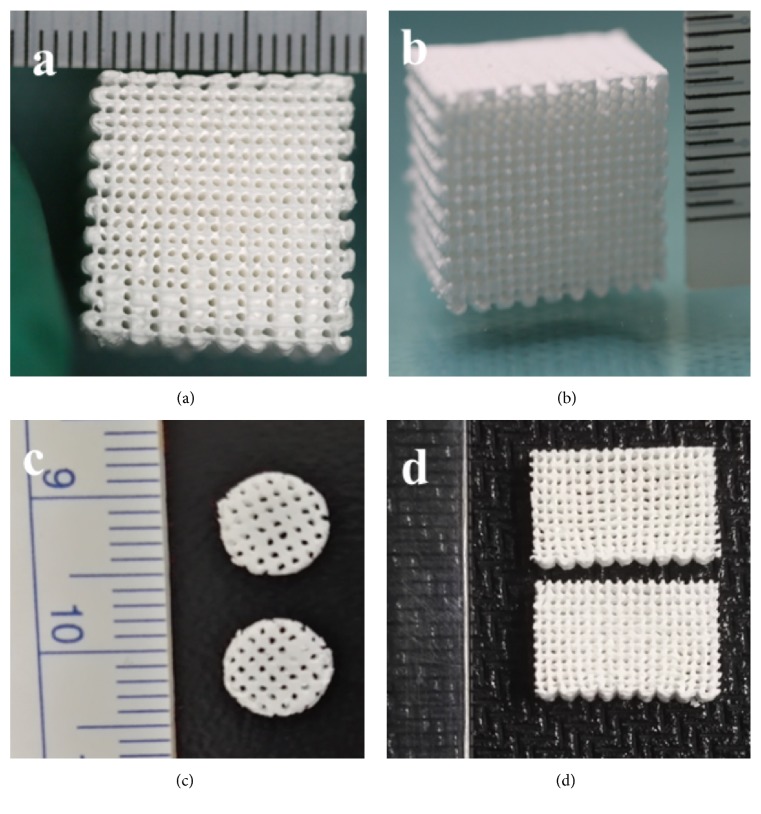
Optical images of PLCL scaffolds.

PLCL scaffold printed by LDM was shown in Figures 1(a) and 1(b). 3D structure of this scaffold can be observed and is similar with the computer-aided designed model. The macropores of the PLCL scaffold were grid-like on the surface of the scaffold. As shown in Figures 1(c) and 1(d), the 3D-printed scaffold was processed and showed various sizes and shapes. Its shape and size not only can be computer-aided designed as needs but also can be processed by hand after molding.

**Figure 2:**
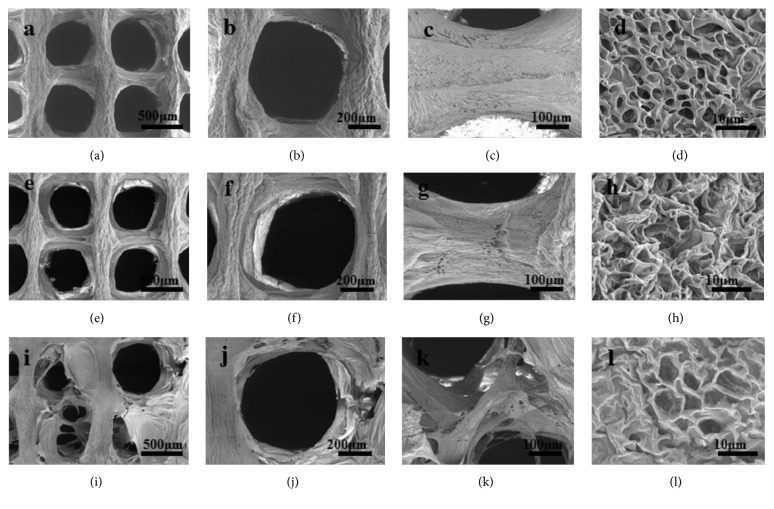
SEM images of PLCL, aPLCL, and PLCL-COLI scaffolds. (a), (b), (c), and (d): different magnifications of PLCL scaffold; (e), (f), (g), and (h): different magnifications of aPLCL scaffold; (i), (j), (k), and (l): different magnifications of PLCL-COLI scaffold.

**Figure 3:**
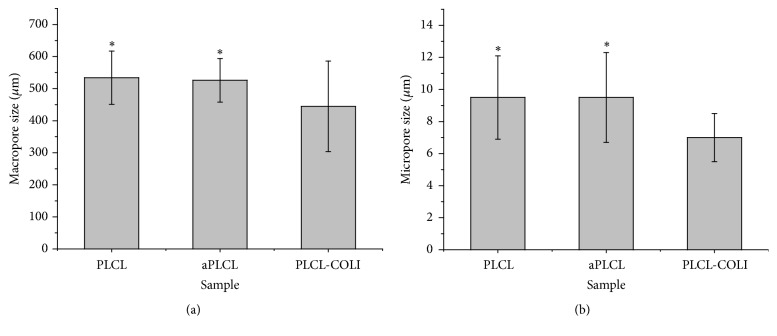
(a) The macropore diameter of PLCL, aPLCL, and PLCL-COLI scaffolds. (b) The micropore diameter of PLCL, aPLCL, and PLCL-COLI scaffolds. ^*∗*^*p* < 0.05.

To observe the morphology and structure of the scaffolds, the scaffolds were cross-sectioned and observed under SEM. As shown in Figures 2(a) and 2(b), similarly round macropores can be observed in the PLCL scaffold, and the diameter of the macropores was 534 ± 84 *μ*m. As shown in Figures 2(a) and 2(c), the printed lines were integrated with each other. And the deposited lines were not as straight as the design; they showed smaller diameter in the middle zones. Under higher magnification, many micropores with diameter between 6.9 *μ*m and 12.1 *μ*m were observed (Figure 2(a)). After the treatment by alkaline, there was no obvious change in the morphology of the macropores, micropores, and deposited lines (Figures 2(e), 2(f), 2(g), and 2(h)). But the diameter of macropores and micropores slightly decreased to 526 ± 68 *μ*m and 9.5 ± 2.8 *μ*m, respectively. Studies showed that the surface of polymeric scaffolds, which were treated by alkaline at proper time periods, showed similar morphology with those of the raw scaffolds. As shown in Figures 2(i), 2(j) and 2(k), flaky layers on the COLI coated PLCL scaffold were observed. We assume that these layers may be COLI, and this phenomenon may be contributed to the high concentration of COLI solution and the rinse process. The diameter of the macropores and micropores in PLCL-COLI scaffold significantly decreased to 445 ± 141 *μ*m and 7.0 ± 1.5 *μ*m (Figure 3), respectively.

Studies have shown that pore morphology, size, and distribution in scaffold played an important role in the growth of cells and the regeneration of tissues.* In vitro*, pores in the biomaterial scaffolds affect scaffolds' topology, which turns to influence cell adhesion, migration, proliferation, and differentiation [31, 32]. The scaffolds printed by LDM not only have regular designed macropores but also have micropores around 10 *μ*m in the printed lines. These unique interconnective micropores created by the gas-solid phase separation may facilitate cell-to-scaffold activity and tissue regeneration [33].

### 3.2. Material Characteristics of the Scaffolds

**Figure 4:**
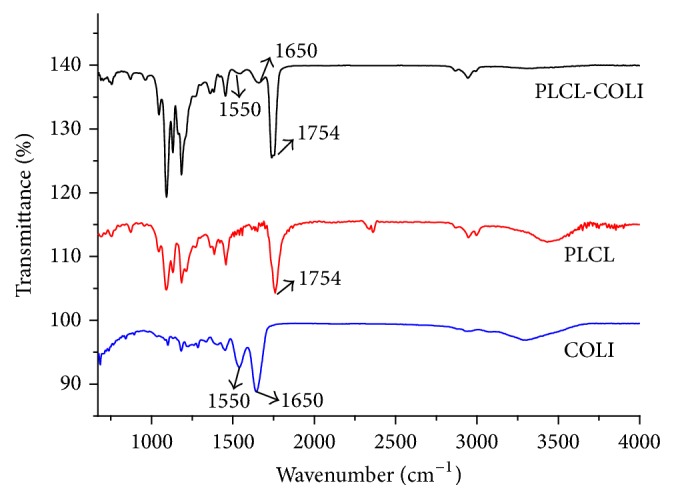
FTIR spectra of PLCL scaffolds, PLCL-COLI scaffolds, and COLI.

In order to confirm that the collagen had been coated on the surface of the PLCL scaffold, PLCL-COLI, PLCL, and COLI were detected by FTIR spectroscopy, and the infrared spectrums were recorded and analyzed (Figure 4). The PLCL infrared spectrum was characterized by the ester A band at 1754 cm^−1^ and ester B band at 1735 cm^−1^. The COLI infrared spectrum was characterized by the amide I (C=O) band at 1650 cm^−1^ and amide II (N-H) band at 1550 cm^−1^. The PLCL-COLI infrared spectrum presented in Figure 4 had both characterized infrared spectrums of PLCL and COLI. From the infrared spectrum analysis, the collagen had been successfully coated on the surface of the PLCL scaffold.

The results of porosity test revealed that the porosity of the scaffolds was up to 80%, and coated COLI did not significantly reduce the porosity of PLCL scaffold (Table 1). Studies have shown that high porosity is conducive to cell adhesion, proliferation, migration, and differentiation. The porosity of 3D scaffold fabricated by conventional 3D printing techniques, such as melt-forming techniques, is generally lower than that of scaffolds printed by LDM. It is difficult to create pores in scaffold by melt-forming techniques. In this experiment, the high porosity was mainly due to phase separation during the scaffold molding process. A large number of interconnected micropores exist in the deposited lines of the scaffold, which significantly increase the porosity of the scaffold.

After COLI coating, the water absorption of PLCL scaffold was improved significantly. It increased from 52 ± 2.7% to 66.9 ± 2.3% (Table 1). The groups of PLCL are mainly methyl and methylene, which are hydrophobic. So the hydrophilicity of PLCL scaffold is relatively low, due to the fact that the COLI consists of a large amount of carboxyl and amino groups, which are excellent hydrophilic and bioactive. The presence of carboxyl and amino groups significantly improves the hydrophilicity of PLCL scaffold. More importantly, when cells adhere to the scaffold surface, the hydrophilic groups may also provide more sites for cell adhesion and facilitate cell behaviors and tissue regenerations [34].

PLCL material is an elastic polymer, which makes the PLCL scaffold flexible and elastic. The PLCL and PLCL-COLI scaffolds could be quickly restored after pressing.

To study the mechanical property of the 3D printed porous scaffolds, compressive tests were performed. Young's modulus of PLCL and PLCL-COLI were 0.32 ± 0.05 MPa and 0.21 ± 0.05 MPa (Table 1), respectively. The mechanical property of PLCL-COLI significantly decreased (*P* < 0.05). Due to alkali treatment, hydrolysis on the surface reduced mechanical properties of PLCL scaffold. When scaffolds are implanted* in vivo*, the mechanical properties of the scaffold play an important role in scaffold-tissue integration [35]. The compressive modulus of human cartilage was between 0.08 MPa and 50 MPa [36]. In this experiment, the modulus of the PLCL-COLI scaffold was 0.21 MPa and, similar to that of native cartilage, this scaffold was suitable in the regeneration of cartilage tissue, especially for elastic cartilage.

### 3.3. Identification of Rabbit Chondrocytes and Biocompatibility of the Scaffolds

**Figure 5:**
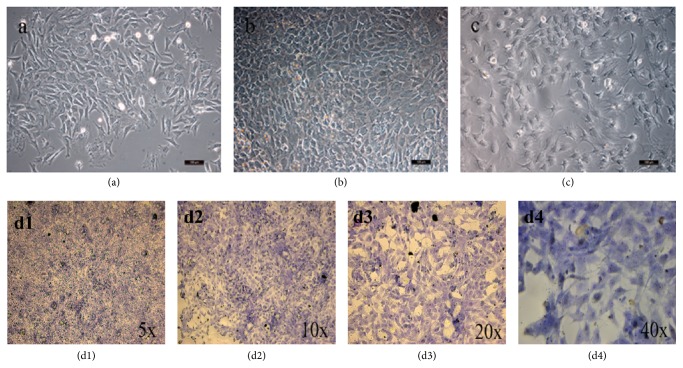
Morphology of chondrocytes under optical microscope (10x). (a) P1, day 6; (b) P1, day 10; (c) P2, day 11. (d1–d4) The results of toluidine blue staining of rabbit articular chondrocytes (P2).

The isolated rabbit chondrocytes were suspended in the media in a spherical shape as shown in Figure 5. After 24 hours, the chondrocytes adhered and became polygonal and round. Subculture was carried out every 72 hours when the cells reached a 90% confluent layer. Subcultured chondrocytes adhered and proliferated within 24 hours, and the cells could reach a 90% confluent layer after 5 to 7 days. The cultured cells were consistent with the morphological performance of chondrocytes under microscope.

As shown in Figures 5(d1)–5(d4), chondrocytes were surrounded by a metachromatic matrix. Blue-violet and heterochromatin granules were observed as well, and a small amount of heterochromatin granules was also found around the cells, which proved that the cells secrete extracellular matrix such as glycosaminoglycans. Under microscope, P2 cells were round and polygonal. It was proved that the cultured cells were chondrocytes.

**Figure 6:**
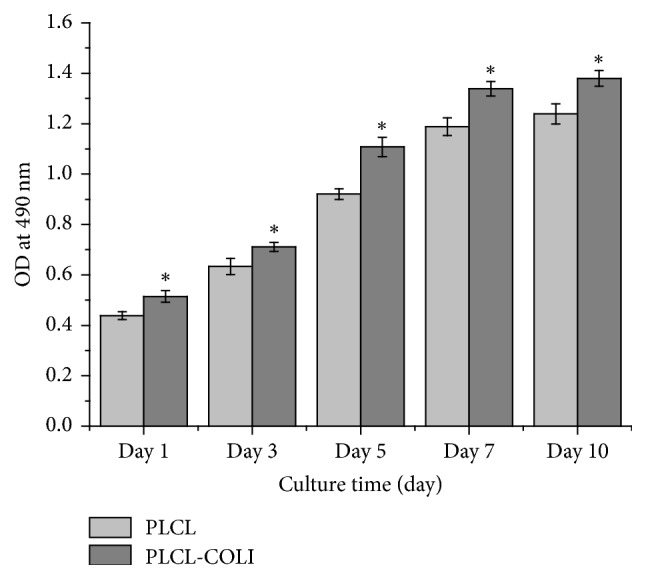
The MTT assay results of rabbit chondrocytes coculturing with PLCL and PLCL-COLI scaffolds, cells on days 1, 3, 5, 7, and 10. ^*∗*^*p* < 0.05.

As shown in Figure 6, after the culture of rabbit chondrocytes on PLCL and PLCL-COLI scaffolds, cell proliferations on days 1, 3, 5, 7, and 10 were characterized by MTT assay. The result showed that the cell number increased with the culture time. At various time points, the cell proliferation on PLCL-COLI scaffold was significantly better than that on PLCL scaffold (^*∗*^*P* < 0.05).

**Figure 7:**
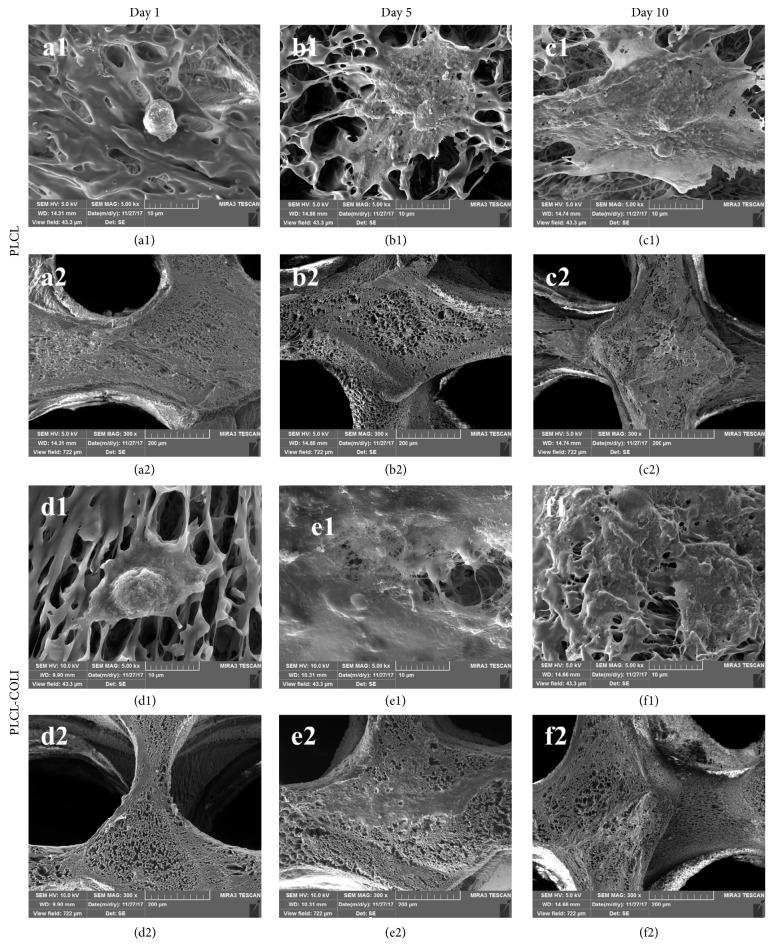
FE-SEM images of rabbit chondrocytes on PLCL and PLCL-COLI. (a1), (b1), and (c1) represent the rabbit chondrocytes on PLCL after being cultured for 1 day, 5 days, and 10 days, respectively; (d1), (e1), and (f1) represent the rabbit chondrocytes on PLCL-COLI after being cultured for 1 day, 5 days, and 10 days, respectively; (a2), (b2), and (c2) represent the lower magnification of (a1), (b1), and (c1), respectively; (d2), (e2), and (f2) represent the lower magnification of (d1), (e1), and (f1), respectively.

The morphology of rabbit chondrocytes on PLCL and PLCL-COLI were studied at different culture time points (1 day, 5 days, and 10 days) by FE-SEM. As shown in Figures 7(a1)–7(c1) and 7(d1)–7(f1), as the culture time lasted longer, the number of cells on PLLA NFS and PLCL-COLI increased. As shown in Figure 7(a1), cell showed ball-shaped morphology on PLCL at day 1. However, cell showed polygonal morphology on PLCL-COLI at day 1. These results indicated that there might be more bioactive cues for cell to attach on the surface of PLCL-COLI. As shown in Figures 7(b1), 7(b2), 7(c1), and 7(c2), cell clusters were observed on PLCL after 5 days and 10 days of culture. As shown in Figures 7(e1), 7(e2), 7(f1), and 7(f2), cell-seeded PLCL-COLI were apparently filled with cell sheets and possibly ECM secreted by the cells.

In cartilage tissue engineering, scaffold is one of core elements which can be temporary as an extracellular matrix to provide skeletal support for tissue regenerations and provide necessary topological cues for cell proliferation, differentiation, nutrient exchange, metabolism, extracellular matrix secretion, and other physiological activities* in vitro* or* in vivo* [37]. Studies suggested that scaffolds made of a single material, such as pure natural or synthetic polymer materials, were difficult to meet the needs of cartilage tissue engineering. And biomimetic composite scaffolds which imitated the composition, structure, and function of natural cartilage tissue were the development tendency in tissue engineering [25, 38]. The 3D scaffolds printed by current technology still have diverse defects. Specifically, composited scaffold consisting of synthetic materials such as PLGA and inorganic nanoparticles such as nHA often maintains mechanical properties and improve hydrophilicity, but its biocompatibility is relatively poor. Composite scaffold consisting of chitosan and inorganic nanoparticles such as nHA have desired biocompatibility, but its mechanical properties are much lower than those of synthetic materials. Therefore, the fabrication of 3D engineered scaffold retaining both desired mechanical properties and biocompatibility is an urgent problem to be solved [24]. Surface modification is a method to improve the biocompatibility of scaffold. There are a variety of surface modification methods, including chemical modification and plasma surface treatment. These methods produce hydrophilic groups such as carboxyl groups, amino groups, or hydroxyl groups on the surface of the scaffolds. They increase the hydrophilicity of the scaffold material and thereby improve the biocompatibility [39]. In this experiment, the PLCL-COLI scaffold was prepared by alkali modification and COLI coating on PLCL scaffold. Although the composite scaffold had relatively lower Young's modulus than that of PLCL scaffold, it meets the need for cartilage regeneration. Importantly, alkali modification and COLI coating significantly improve the biocompatibility of PLCL scaffold. There are still some problems to be solved in this study, such as oversized aperture, shrinkage, and partial occlusion. To solve these problems, modeling, printing parameters, and modification method are needed to be refined. Another prominent problem is the poor combination of COLI and PLCL. However, this PLCL-COLI shows its own advantages in tissue engineering, such as the designed and controlled structure, high porosity and hydrophilicity, elastic property, and good biocompatibility. The PLCL-COLI scaffold may work as a graft in cartilage repair.

## 4. Conclusions

In this study, 3D porous PLCL scaffolds were fabricated by the LDM technology, and PLCL-COLI scaffold was prepared by alkali treatment and COLI coating on the PLCL scaffold. These scaffolds had designed macropores, interconnected micropores, and high porosity. After COLI coating, the hydrophilicity and cell compatibility of PLCL were enhanced. Although the mechanical property of PLCL-COLI slightly decreased after alkali treatment, its Young modulus meets the need for cartilage regeneration. The PLCL-COLI scaffold is expected to be one of the ideal cartilage tissue scaffolds by further modeling, regulating parameters, improving modification method, and strengthening the combination of collagen and PLCL.

## Figures and Tables

**Table 1:** Porosity, hydrophilicity, and compressive Young's modulus of PLCL and PLCL-COLI scaffolds (^*∗*^*P* < 0.05).

Scaffold	1	2	3
	Porosity (%)	Hydrophilicity (%)	Young's modulus (MPa)
PLCL	86.7 ± 1.8	52.0 ± 2.7	0.32 ± 0.05^*∗*^
PLCL-COLI	84.7 ± 1.7	66.9 ± 2.3^*∗*^	0.21 ± 0.05
